# Peripheral blood immune inflammatory markers in neuromyelitis optica spectrum disorders

**DOI:** 10.3389/fneur.2025.1690767

**Published:** 2025-11-06

**Authors:** Bin Wu, Fangzheng Cao, Mengying Mao, Xiaoli Gong, Bin Xu

**Affiliations:** 1 The Second Clinical Medical College of Zhejiang Chinese Medical University, Hangzhou, China; 2 Department of Neurology, The Second Affiliated Hospital of Zhejiang Chinese Medical University, Hangzhou, China; 3 Department of Neurology, Zhejiang Provincial People's Hospital, Hangzhou, China

**Keywords:** optica spectrum disorder, peripheral blood immune inflammatory markers, lymphocyte-to-monocyte ratio, neutrophil-to-lymphocyte ratio, platelet-to-lymphocyte ratio

## Abstract

Neuromyelitis optica spectrum disorder (NMOSD) is an autoimmune neuroinflammatory disease with high relapse risk and cumulative neurological disability. Identifying, providing early warning, and reproducible monitoring of disease progression and therapeutic efficacy in peripheral blood biomarkers is crucial for clinical management and personalized treatment. Numerous studies have investigated the relationship between peripheral blood immune-inflammatory markers including cytological ratios, cytokines, complement components and NMOSD disease activity, relapse risk, and long-term outcomes, aiming to evaluate their potential application in clinical prognostic assessment and treatment monitoring. Meta-analyses have shown that the neutrophil-to-lymphocyte ratio (NLR) is significantly elevated in patients with NMOSD compared with healthy controls (mean difference (MD) approximately 1.04, 95% CI 0.76–1.32; high heterogeneity). The NLR is associated with relapse risk and disability (EDSS ≥4) (OR for relapse, approximately 1.33–2.14; OR for EDSS ≥ 4, approximately 1.23–1.43), supporting the potential clinical application of peripheral blood Immune inflammatory markers in NMOSD. This review summarizes the current evidence for peripheral blood inflammatory markers in NMOSD, focusing on their clinical application.

## 1 Introduction

Neuromyelitis optica spectrum disorder (NMOSD) is a rare but severe autoimmune inflammatory demyelinating disease of the central nervous system (CNS) (1, 2), characterized by high relapse rates, cumulative disability, and increased mortality (3, 4). It is primarily mediated by pathogenic immunoglobulin G antibodies targeting aquaporin-4 (AQP4), the most abundant water channel protein in astrocytic end-feet (5, 6). The disease predominantly affects the optic nerves and spinal cord (7–9), and less frequently the brainstem, leading to severe clinical manifestations such as recurrent optic neuritis (10), transverse myelitis (11), and brainstem encephalitis (12). Epidemiology of NMOSD is approximately 1 per 200,000 individuals (13). The mean age of NMOSD onset is between 30 and 40 years with a striking female predominance, and the female-to-male ratio is approximately 9–11:1 (14, 15).

The immunopathogenesis of NMOSD is complex and incompletely understood. Current evidence indicates that pathogenic AQP4-IgG antibodies initiate a cascade of complement-mediated astrocytic injury, inflammation, and demyelination, leading to irreversible neuronal and axonal damage. Pro-inflammatory cytokines and innate immune cells, including neutrophils, eosinophils, and natural killer (NK) cells, have been implicated as critical mediators in this process (16). The discovery of AQP4-IgG in 2005 represented a major breakthrough, allowing NMOSD to be distinguished from MS with high specificity and transforming diagnostic and therapeutic approaches (7, 17). The International Panel for NMO Diagnosis (IPND) revised the diagnostic criteria in 2015 to incorporate serological markers and clinical phenotypes, providing a standardized framework for global diagnosis (7).

While autoantibody-mediated astrocyte attack has been identified as a core mechanism, increasing evidence suggests that dysregulation of the peripheral immune system plays a crucial role in disease development, progression, and clinical outcome. Increasing evidence highlights the role of cytokines and peripheral immune cell activity in disease development and progression. Elevated levels of interleukin-6 (IL-6) (18), interleukin-17 (IL-17) (19, 20), and activated immune cell subsets have been identified as mediators of central nervous system inflammation in NMOSD (21). These findings have stimulated interest in peripheral blood inflammatory markers as convenient, non-invasive indicators of systemic immune responses. Comprehensive hematologic measures such as the neutrophil-to-lymphocyte ratio (NLR) (22), platelet-to-lymphocyte ratio (PLR) (23), and monocyte-to-lymphocyte ratio (MLR) may provide a comprehensive reflection of the immune-inflammatory balance (24). Unlike absolute cell counts, these ratios capture the dynamic interactions between immune cell subsets. These peripheral changes not only contribute to disease pathophysiology, diagnosis, and differential diagnosis, but also provide promising biomarkers for monitoring disease activity, predicting relapse risk, and assessing therapeutic efficacy (25). Given the challenges of repeated cerebrospinal fluid sampling and the need for noninvasive monitoring tools, identifying reliable peripheral biomarkers is particularly important for NMOSD.

This review synthesizes current evidence on peripheral blood inflammatory markers in NMOSD, focusing on their clinical applications. By integrating findings from cellular immunology, cytokine profiling, and complement biology, we aim to provide a structured overview of how these markers reflect and potentially influence disease activity and prognostic value in NMOSD.

## 2 Peripheral blood inflammatory indexes in NMOSD

### 2.1 Neutrophil-to-lymphocyte ratio (NLR)

The neutrophil-to-lymphocyte ratio (NLR) is defined as the absolute neutrophil count divided by the absolute lymphocyte count in peripheral blood (NLR = N/L) (23). NLR reflects systemic inflammation and provides a more sensitive indicator than total leukocyte or subtype counts. Elevated NLR has been associated with disease severity in malignancies (26, 27), endocrine disorders, and acute coronary syndromes (28, 29). NLR is also associated with autoimmune diseases, including multiple sclerosis (MS) (30), systemic lupus erythematosus (SLE) (31), Behçet's disease, primary Sjögren's syndrome (pSS) (32), and inflammatory bowel disease (IBD) (33). NLR is also associated with disease severity in NMOSD (34). A retrospective study showed that NLR levels were significantly higher in NMOSD patients than in healthy controls (*p* < 0.001) (34). Another study compared data from 259 newly diagnosed NMOSD patients with 169 healthy controls undergoing physical examinations during the same period showing the same result (35). Furthermore, NLR levels were higher in patients with acute exacerbations than in those in remission (*p* < 0.001). NLR was positively correlated with increased disability from acute exacerbations [ΔEDSS (*r* = 0.301, *p* = 0.016)], suggesting that NLR may be a useful indicator for assessing NMOSD disease activity. Furthermore, NLR may reflect the severity of neurological dysfunction. In first-episode NMOSD patients, NLR may be an independent risk factor for the severity of neurological deficits. Among NMOSD patients, those with higher NLR had greater severity of neurological deficit at onset than those with lower NLR (*P* < 0.001); patients with severe neurological deficit had a higher NLR at onset than those with mild to moderate neurological deficit (*P* < 0.001). Both univariate logistic regression analysis (OR 1.180, 95% CI 1.046–1.331, *P* = 0.007) and multivariate logistic regression analysis (OR 1.146, 95% CI 1.003–1.308, *P* = 0.044) showed a positive correlation between NLR and the severity of neurological deficit at onset in NMOSD patients, with the area under the receiver operating characteristic curve was 0.687. Therefore, NLR is an independent risk factor for the severity of neurological deficits in patients with first-episode NMOSD (36). NLR has been identified as an independent prognostic marker in newly diagnosed NMOSD, significantly correlating with poor recovery. A retrospective study of 324 newly diagnosed NMOSD patients showed that high NLR was associated with significantly higher EDSS scores and recurrence rates. Multivariate analysis confirmed NLR as an independent predictor of recurrence (HR = 1.07, 95% CI: 1.03–1.10, *P* = 0.001) and poor recovery (HR = 1.08, 95% CI: 1.04–1.11, *P* = 0.001) (36). In the Xie et al. cohort, an NLR of 2.38 was used to predict relapse (sensitivity 81.8%, specificity 64.7%; AUC = 0.781); the optimal cutoff for predicting poor recovery was 2.63 (sensitivity 76.3%, specificity 68.0%; AUC = 0.746) (36). If the goal is to predict greater disability (e.g., increased EDSS score) or as a more conservative marker of “high risk,” some studies or meta-analyses have reported thresholds of 3.9–4.52 (sensitivity and specificity varies widely across studies). These findings suggest that the NLR correlates with NMOSD disease activity and is a valuable prognostic marker for NMOSD. However, its clinical utility is limited by its specificity, as the NLR may be confounded by systemic infection (37) or glucocorticoid therapy (38), both of which significantly increase neutrophil counts. Therefore, while the NLR may be most useful as an initial screening tool or for rapid risk stratification, confirmatory evaluation with other clinical and laboratory parameters is crucial before guiding treatment decisions.

### 2.2 Monocyte-to-lymphocyte ratio (MLR)

The monocyte-to-lymphocyte ratio (MLR) is calculated as the absolute monocyte count divided by the absolute lymphocyte count in peripheral blood (MLR = M/L) (39). MLR is recognized as an inflammatory marker and has been increasingly applied in cardiovascular diseases (40, 41), where it has demonstrated prognostic significance. Elevated neutrophil and monocyte counts, combined with reduced lymphocyte counts, are associated with the promotion of atherosclerosis (42, 43). Among these cells, monocytes play a central role by migrating into cardiac tissue, differentiating into macrophages, and secreting inflammatory cytokines, thereby contributing significantly to disease progression. Evaluation of monocyte activity, therefore, provides valuable prognostic information.

Beyond cardiovascular disorders, MLR has also been implicated in central nervous system demyelinating diseases and other autoimmune conditions (44, 45). As for NMOSD, a retrospective study comparing 72 NMOSD patients with 72 healthy controls found that serum MLR levels were significantly higher in patients (46). Regarding the prediction of NMOSD disease, recent prospective or retrospective cohorts with smaller sample sizes used receiver operating characteristic (ROC) analysis to identify an MLR of 0.34 as the optimal cutoff for predicting 12-month relapse (AUC ≈ 0.76; sensitivity ≈ 54.5%, specificity ≈ 95.3%) (47). This suggests that the MLR has high specificity but moderately low sensitivity for predicting relapse. Another retrospective study comparing the clinical data of 9 AQP4-positive NMOSD patients and 42 MS patients concluded that MLR can predict the 1-year disability level of children with NMOSD (48). The above research results require studies with larger sample sizes to be more convincing. Although the evidence for MLR is less robust than that for NLR or PLR (Table 1), it shows potential for predicting short-term relapse of NMOSD with a high positive predictive value and could serve as an aid in making decisions about accelerating or extending immunosuppressive therapy in established patients.

**Table 1:** Comparative analysis between biomarkers.

**Biomarker**	**Study design**	**Sensitivity (%)**	**Specificity (%)**	**AUC**	**Outcome**	**Sample size**	**Limitation**	**Clinical Utility**	**References**
NLR	Retrospective cohort	81.80%	64.70%	0.781	Relapse	162 Low-NLR group with NMOSD VS 162 High-NLR group with NMOSD	Small sample size, single center and missing follow-up data	Prediction of relapse	Xie et al. (36)
NLR	Retrospective cohort	76.30%	68.00%	0.746	Poor recovery (EDSS ≥ 4)	162 Low-NLR group with NMOSD VS 162 High-NLR group with NMOSD	Small sample size, single center and missing follow-up data	Prediction adverse outcomes	Xie et al. (36)
MLR	Retrospective cohort	54.50%	95.30%	0.76	Recurrence within 12 months	54 NMOSD patients and 54 MS patients and 54 Healthy individuals	Small sample size, short follow-up period	Prediction of relapse	Fang et al. (47)
MLR	Retrospective cohort	NR	NR	NR	1-year disability in pediatric NMOSD	8 NMOSD patients and 28 MS patients	Small sample size, single-center design, The time limit for data collection	Predict 1-year disability in pediatric NMOSD	Devlin et al. (48)
PLR	Retrospective cohort	77.80%	88.90%	0.815	Active vs. remission	72 NMOSD patients and 72 healthy controls	Retrospective design, single-center design, potential selection bias	Distinguish phase	Yan et al. (46)
PLR	Retrospective cohort	65%	90.48%	0.8116	Severity (EDSS ≥ 4)	72 NMOSD patients and 72 healthy controls	Retrospective design, single-center design, potential selection bias	Assessing disability severity (EDSS)	Yan et al. (46)
CRP	Retrospective cohort	NR	NR	NR	EDSS	NMOSD (*n* = 106), MS (*n* = 87), PD (*n* = 79) and healthy controls (*n* = 80)	Relatively small sample size, bias of retrospective study	Assessing disability severity (EDSS)	Shu et al. (76)
IL-6	Retrospective cohort	NR	NR	NR	Active vs. remission	26 NMOSD patients and 12 healthy controls	Small sample size	Assessment of disease activity	Haramati et al. (97)
IL-6	Retrospective cohort	NR	NR	NR	Recurrence within 2-year follow-up period	20 patients with relapsed remission stage of NMOSD and 20 healthy controls	Small sample size	Prediction of relapse	Barros et al. (98)
IL-17	Retrospective cohort	NR	NR	NR	Concentrations of some cytokines during relapse	14 NMOSD patients and 20 MS patients and 16 Healthy individuals	Small sample size	NR	Li et al. (100)
C3a	Prospective cohort	94.7%	90.3%	0.9479	Recurrence within 2-year follow-up period	19 NMOSD patients and 35 MS patients and 40 Healthy individuals	Differences in disease treatment (MS vs. NMOSD) and small sample size	Assessment of disease activity	Nytrova et al. (106)

NR, not reported.

### 2.3 Platelet-to-lymphocyte ratio (PLR)

The platelet-to-lymphocyte ratio (PLR) is calculated as the absolute platelet count divided by the absolute lymphocyte count in peripheral blood (PLR = P/L) (49). PLR is recognized as a reliable marker of systemic inflammation (49). In oncology, PLR has been shown to have prognostic value, as tumor-associated inflammation (50) and inflammatory mediators, including cytokines and chemokines, influence disease outcomes (51, 52). By reflecting the systemic inflammatory burden, PLR provides an indirect measure of the extent of tumor-associated inflammation and may, in some contexts, outperform the neutrophil-to-lymphocyte ratio (NLR) in predicting disease severity (53, 54).

Beyond cancer, numerous studies have demonstrated the relevance of PLR across immune-mediated, metabolic, thrombotic, and neoplastic disorders (55). It has been identified as a predictor of severe disease progression, concurrent infections, aneurysmal coronary artery disease, and outcomes in inflammatory rheumatic diseases (56, 57). These findings highlight PLR as a broadly applicable inflammatory biomarker.

In NMOSD, PLR correlates positively with disease severity, as reflected by baseline EDSS scores (58). A retrospective analysis of 72 NMOSD patients and 72 healthy controls showed a significant correlation between NMOSD severity and PLR (*P* = 0.000) on univariate analysis, and multivariate analysis confirmed the significance of PLR (*P* = 0.000), indicating that serum PLR may independently contribute to the assessment of NMOSD severity (46). In addition, there was a positive correlation between PLR and baseline EDSS score, suggesting that serum PLR can serve as an independent indicator of NMOSD severity (46). These findings suggest that serum PLR can serve as a practical and cost-effective biomarker for assessing NMOSD severity and monitoring recurrence risk.

In some studies, with combining with the blood-brain barrier marker Qalb, a PLR ≥ 113 was significantly associated with moderate to severe NMOSD (EDSS ≥ 4) in the acute phase, serving as an independent risk factor in this cohort (59). PLR has also been discovered as superior specificity for the risk of disease severity compared to the NLR and is also helpful in distinguishing NMOSD from other demyelinating diseases. The PLR can be affected by thrombocytosis (60), infection (61), bleeding, or antiplatelet medications (62). Therefore, when using PLR in the clinical application of NMOSD, these interfering factors need to be considered. Future multicenter, prospective studies are needed to further enhance the role of PLR in assessing disease activity and predicting outcomes in NMOSD.

## 3 Other inflammatory factors and NMOSD

### 3.1 C-reactive protein (CRP)

C-reactive protein (CRP) is an acute-phase protein predominantly synthesized by the liver in response to cytokine signaling at sites of tissue injury or infection. It plays a central role in promoting acute inflammatory responses and has long been considered a component of the innate immune system, functioning as a pattern recognition receptor that binds phosphocholine ligands (63–65). Its rapid synthesis and short half-life make CRP a practical serological marker for the acute phase of inflammation, allowing both diagnostic assessment and monitoring of treatment response (66).

CRP levels were found to be associated with cancer risk, viral infections, and cardiovascular disease (67–69). It also serves as a biomarker for disease activity in various immune-mediated conditions, including Crohn's disease (70), Sjögren's syndrome (71), systemic lupus erythematosus (72), and rheumatoid arthritis (73). Besides, elevated CRP levels are established independent predictors of cardiovascular events (44, 74), including atherosclerosis, congestive heart failure, atrial fibrillation, myocarditis, and outcomes following heart transplantation.

Cross-sectional and retrospective cohort reports indicate that serum CRP levels are elevated in patients with active NMOSD (75), and in some cohorts, are positively correlated with acute disability scores such as EDSS (76). These studies suggest that CRP may reflect acute inflammatory burden or a concurrent systemic inflammatory state. On the other hand, several recent multi-marker and multi-omics comparative studies have demonstrated that elevated CRP levels are not consistently present in all cohorts, and its independent predictive power for relapse risk or long-term disability progression is often attenuated or lost in multivariate analyses (47, 77, 78). This discrepancy may be due to CRP's high sensitivity to various nonspecific stimuli such as infection, trauma, and concurrent autoimmune diseases (75), and its susceptibility to comorbidities (79) and treatments (e.g., corticosteroids) (80), leading to confounding bias in cohorts that haven't rigorously excluded concurrent infections or systemic diseases. Furthermore, many studies have employed cross-sectional designs or limited sample sizes, and lack consistency in sample collection or processing and testing methods, comparability and measurement accuracy across studies are limited. Limiting the value of CRP in independent prognostic models. Based on current evidence, CRP may not be supported as a standalone or primary diagnostic or prognostic biomarker for clinical decision-making in NMOSD. Its integration with other peripheral blood inflammatory markers may enhance the assessment of disease severity and prognosis, particularly in immune-mediated and acute inflammatory states. Further research and trials, such as prospective cohort studies, are needed to further clarify its potential value.

### 3.2 IL-6 (interleukin-6)

Interleukin (IL) family cytokines primarily regulate cell proliferation, differentiation, and activation within immune and inflammatory responses. Specifically, IL-1, IL-6, and IL-17 are key mediators in the pathogenesis of autoimmune neuroinflammatory disorders resulting from aberrant immune responses in the nervous system (81). IL-1 contributes to immune cell activation, promotes pro-inflammatory cytokine production, and disrupts the integrity of the blood-brain barrier (82). IL-17, a potent pro-inflammatory cytokine secreted by Th17 cells, critically recruits immune cells to sites of inflammation (83). IL-6 is produced by macrophages, dendritic cells, and B cells, and is essential for differentiating naïve T cells into Th17 cells (84). IL-6 contributes to neuroinflammation, enhances antibody production by activated B cells, and facilitates the differentiation of naïve T cells into Th17 cells (85).

IL-6 is a key regulator of acute and chronic inflammation and hematopoiesis and is involved in inflammation, antigen-specific immune responses, host defense, hematopoiesis, and the synthesis of acute phase proteins (86). Within adaptive immunity, IL-6 promotes antibody production and supports effector T cell development (87). Aberrant IL-6 expression or signaling contributes to numerous diseases, including inflammatory and lymphoproliferative disorders such as giant cell arteritis, multiple myeloma, rheumatoid arthritis, and systemic lupus erythematosus (SLE) (88). After injury, neurons, astrocytes, microglia, and endothelial cells secrete IL-6, leading to elevated cerebrospinal fluid (CSF) IL-6 levels in neuroinflammatory diseases (88). In NMOSD patients, CSF IL-6 levels are typically elevated and correlate with disease severity (89). Studies have shown that the role of IL-6 in the pathogenesis of NMOSD is mediated by increased antibody production and antigen presentation, increased plasmablast and B cell levels, and decreased regulatory B cells (90). Increasing evidence indicates that NMOSD patients exhibit higher serum IL-6 levels compared with healthy individuals or patients with other diseases. Uzawa et al. reported elevated serum IL-6 in NMOSD patients compared with individuals with non-inflammatory neurological diseases (ONNDs) (91), a finding corroborated by Wang et al., who observed higher plasma IL-6 in NMO patients vs. healthy controls (92, 93). NMOSD patients exhibit markedly elevated CSF and serum IL-6 levels, and IL-6 inhibition has been shown to improve disease management (94, 95). For example, the IL-6 receptor antibody satralizumab has been approved for the treatment of NMOSD, but some neutrophils have decreased after treatment with this drug (96), suggesting that this drug has an effect on neutrophil activity. A cohort study comparing serum IL-6 levels in 26 NMOSD patients during relapse and remission showed that IL-6 levels were significantly elevated in NMOSD patients during relapse compared with remission (*n* = 19, 8.39 ± 10.84 pg/ml vs. *n* = 19, 2.02 ± 2.27 pg/ml, *p* = 0.001) and in HCC patients (*n* = 16, 1.23 ± 1.18 pg/ml, *p* < 0.01). This suggests that IL-6 levels are significantly elevated in NMOSD patients during relapse (97). Furthermore, elevated IL-6 levels correlate with relapse severity. Therefore, serum IL-6 levels can serve as a biomarker for NMOSD disease activity and IL-6 detection helps in disease stratification and personalized medication decision making. Regarding the role of IL-6 levels in the prognosis of NMOSD patients, studies have shown that IL-6 levels produced during remission can predict the severity of NMOSD relapse (98). However, this study involved a small sample size. Therefore, the role of IL-6 levels in NMOSD prognosis requires further clarification in multicenter, large-sample prospective studies. Future research will demonstrate significant potential for combined modeling of IL-6 with other peripheral blood inflammatory markers.

### 3.3 IL-17 (interleukin-17)

Cytokines are low molecular weight proteins that regulate immune and inflammatory responses. Cytokines play a crucial role in regulating neuroinflammatory responses by recruiting and activating different types of cells (99). IL-17 is a potent proinflammatory cytokine produced by Th17 cells that plays a crucial role in recruiting immune cells to inflammatory sites (83). It is well-known that IL-17 signaling is crucial for the development of autoimmune diseases and is involved in biological processes such as neutrophil infiltration into the CNS and promotion of neuroinflammation (100). Studies have shown that NMOSD patients exhibit activated IL-17 signaling (101). Compared with HCC, NMOSD patients have increased expression of the cytokine IL-7 in peripheral blood (102). A large number of studies have confirmed that certain cytokines or chemokines and related molecules such as IL-17 are associated with the clinical activity (102) and long-term prognosis of NMOSD. A meta-analysis included 17 studies reporting IL-17 levels in NMOSD patients and controls. The results showed that IL-17 levels in NMOSD patients were higher than those in the control group [0.87, (0.42, 1.33), *P* < 0.001], but there was significant heterogeneity, which was considered to be caused by the use of a random effects model. Accumulating evidence suggests that IL-17 is crucial for disease activity and adverse outcomes in AQP4-IgG-positive NMOSD (103), but evidence on their independent predictive value remains limited. Multiple cross-sectional and case-control studies have detected elevated IL-17A levels in serum and cerebrospinal fluid during relapse or active phases. Further meta-analyses and reviews have summarized these cross-sectional observations, suggesting that the proportion of Th17 cells and Th17 core cytokines are generally higher in NMOSD than in healthy controls, and are often associated with active disease (102). These evidences support the IL-17 or Th17 pathway as a candidate prognostic marker and “biomarker of treatment response,” but more adequate longitudinal cohort data are needed to demonstrate that baseline or early follow-up IL-17 levels can independently predict future outcomes such as relapse rate, disability progression, or death. In conclusion, IL-17 plays an important role in disease activity and worse clinical outcomes in NMOSD. However, its use as an independent prognostic marker requires further investigation.

### 3.4 Complement system

The complement system, a component of the innate immune system, facilitates pathogen clearance through antibody and phagocyte-mediated mechanisms. While complement activation is generally protective, excessive or dysregulated activation can cause tissue damage (104). Relevant studies have shown that there is a correlation between complement components and the ratio of neutrophils to lymphocytes. A retrospective study analyzing blood indicators, complement information and other related data of 75 healthy adolescents showed that there is a positive correlation between NLR and C3a and C4 levels (105). Plasma C3a levels correlate with NMOSD activity, as measured by the EDSS (106), highlighting the potential of C3a as a biomarker for disease activity (107). Recent evidence indicates that C3a mediates microglial activation and contributes to early CNS pathology in NMOSD (108). Accordingly, C3a levels may serve as an indicator of disease activity in NMOSD. Peripheral blood complement products play an important role in reflecting disease activity and predicting clinical outcomes (109). Multiple clinical cohorts and systematic reviews have demonstrated that activated complement fragments, such as Ba, C3a, C5a, and the terminal complex sC5b-9 are often significantly elevated during relapses or periods of increased disease activity (77). More significance is the association of complement markers with disease severity or short-term outcomes in several studies: serum or cerebrospinal fluid sC5b-9 levels are positively correlated with the EDSS score and higher sC5b-9 or Ba levels are associated with an increased risk of near-term relapse (110), a pattern replicated in multicenter and single-center samples. However, research on the relationship between complement levels and NMOSD disease activity and prognosis in clinical practice is lacking. Existing studies also have limitations, requiring further refinement, such as expanding sample sizes and conducting prospective studies. Additionally, studies have combined complement C3 and C3a with the neutrophil ratio for some inflammatory and disease activity assessments (111). Therefore, combining complement levels with conventional peripheral inflammatory markers for joint modeling may be a promising approach for future clinical application in NMOSD patients, thereby transforming complement markers into reliable prognostic biomarkers and influencing clinical decision-making.

### 3.5 Follicular helper T cells (Tfh)

Tfh cells are a novel type of CD4+ T cell that play a role in assisting B cell activation and maturation (112). In recent years, the role of Tfh cells in NMOSD has garnered increasing attention. Studies in an animal model have shown that Tfh is involved in the pathogenesis of neurological autoimmune diseases (113). Existing studies have shown that the proportion of peripheral blood Tfh cells is significantly elevated in patients with acute-phase NMOSD who are serum AQP4 antibody-positive (114). This increase correlates with acute disease activity, suggesting that the Tfh ratio is involved in the pathogenesis of NMOSD. Furthermore, a study comparing blood samples from 36 patients in the acute and recovery phases of NMOSD, 20 patients with other non-inflammatory neurological diseases (ONND), and 20 age- and sex-matched healthy volunteers showed that the proportion of peripheral blood Tfh cells in acute-phase NMOSD patients was positively correlated with EDSS scores (*r* = 0.596, *P* < 0.001), suggesting that Tfh cell levels may serve as a biomarker for disease severity (115). The current study still has a small sample size, and further multicenter, large-sample studies are needed in the future to further support the relationship between Tfh and NMOSD disease activity. However, their clinical application in predicting prognosis requires further investigation.

### 3.6 Plasmablasts

Plasmablasts are intermediate-stage cells that differentiate from B cells during the immune response (116). Plasmablasts are involved in disease pathogenicity and immune regulation. Analyzing plasmablast levels may enable the diagnosis and assessment of the disease status of autoantibody-related neuroimmune diseases, such as NMOSD (117). A study analyzing blood samples from patients with NMOSD and other neuroinflammatory diseases, as well as healthy individuals, demonstrated an increased frequency of plasmablasts in NMOSD patients compared with patients with relapsing-remitting multiple sclerosis and healthy individuals (118) (Table 2). Another study also suggests a direct correlation between plasmablast levels and AQP4-IgG production in NMOSD (119). These results may suggest a correlation between plasmablast levels and disease activity in NMOSD. But, the current study still involves a small sample size, and future large-sample, multi-center prospective studies are needed to further clarify the association between plasmablasts and NMOSD disease activity. However, research on the clinical application of plasmablasts in NMOSD prognosis is currently lacking and warrants further investigation. Furthermore, various B cell subsets involved in downregulating excessive immune and inflammatory responses have protective effects. These B cell subsets are often referred to as regulatory B cells (Bregs). Bregs play a crucial role in several immune diseases, and Breg subsets may play a complex role in the pathogenesis of NMOSDs (120). Current research has shown that Breg numbers or function are decreased in NMOSD patients, suggesting a possible correlation between Bregs and disease activity (90). However, research in this area remains limited, and further study is needed to clarify this issue. Current research on the role of Bregs in NMOSD prognosis is insufficient, and further research is needed to explore this relationship and inform clinical practice in NMOSD.

**Table 2:** Trends in biomarker levels.

**Biomarker**	**Remission (vs. healthy individuals)**	**Relapse (vs. remission)**	**References**
NLR	↑	↑	Benetou et al. (127)
MLR	NR	↑	Fang et al. (47)
PLR	NR	↑	Fang et al. (47)
CRP	NR	↑	Shu et al. (76)
IL-6	↑	↑	Haramati et al. (97)
IL-17	NR	↑	Li et al. (100)
C3a	NR	↑	Nytrova et al. (106)
C3/C4	NR	↓	Rodin and Chitnis (97)
Tfh	↑	↑	Yang et al. (115)
Plasmablasts	—	↑	Akatani et al. (90)

NR, not reported; —, almost the same; ↑, increase; ↓, decline.

## 4 Conclusion

Peripheral blood inflammatory markers, including NLR, PLR, MLR, IL-6, IL-17, CRP, Plasmablasts, Tfh and the complement system, have become important tools for assessing disease activity and prognosis in NMOSD. In particular, NLR and PLR, according to existing research (34–36), have been shown to be significantly correlated with disease severity, relapse risk, and functional outcome, reflecting the systemic immune-inflammatory state and the underlying pathophysiology involving neutrophil-, platelet-, and monocyte-mediated processes, and therefore have relatively high clinical practical application value. However, more research is needed on MLR, IL-6, IL-17, CRP, Plasmablasts, Tfh and complement system to further clarify the reliability of their application value in clinical practice.

Timely assessment of disease activity and prediction of relapse are critical for NMOSD management. Although traditional methods such as imaging and serological tests can provide important information, for example, magnetic resonance imaging (MRI) is crucial in the early identification of NMOSD and guiding immediate acute treatment decisions (121). It is currently the standard for monitoring NMOSD lesions, especially in the diagnosis of NMOSD seronegative cases (122). It plays an increasingly important role, but they are often costly, invasive or time-consuming. In contrast, inflammatory markers detected by routine blood cell counts are simple, reproducible and cost-effective, and can dynamically monitor immune status and disease progression. Based on current research, routine clinical measurements such as NLR, PLR, MLR, CRP, complement C3/C4, and, if available, serum IL-6 are recommended upon diagnosis or initial visit (47). These measurements can serve as baseline references for follow-up. In the event of suspected or confirmed relapse, NLR, PLR, MLR, and CRP should be immediately repeated, and serum IL-6 and complement activation products should be measured, if possible, to assess inflammation and adjust clinical treatment (97). For patients at risk of relapse or who have recently discontinued medication, the use of NLR, PLR, and MLR as inexpensive and easily available routine follow-up markers for NMOSD disease assessment may be a good option. If used to predict short-term relapse, such as MLR, remeasurement can be performed 3–6 months after the initial hospitalization or baseline, and clinical judgment can be used to consider intensification or adjustment of maintenance therapy (47). Furthermore, when using anti-complement therapies such as IL-6R inhibitors or eculizumab, monitoring of relevant pathway markers such as IL-6, CRP, and complement products can help assess drug effects and complication risks (95), but understanding the effects of the medication itself on these markers is crucial. However, further in-depth research is needed on the specific application of these peripheral blood inflammatory indicators in clinical practice in the future. It is important to note that there is currently a lack of research directly comparing the effects of MRI and peripheral blood inflammatory markers on NMOSD disease outcomes (123). Future research should further improve direct comparisons of the two to further conclude that peripheral blood inflammatory markers may become a faster and cheaper method.

Each marker also has intrinsic strengths and weaknesses, and using a single parameter may provide an incomplete assessment. Existing studies on NLR, PLR, MLR, CRP, IL-6, IL-17, or complement C3 and C4 have mentioned that “multiple indicators have the potential to improve predictive or differential value in NMOSD” (47, 77, 123). However, there are few validated, clinically applicable combined prognostic scores or threshold systems (124). This is a clear gap in the current literature, suggesting that future research needs to improve multicenter, large sample size, and combined modeling with other indicators to build a comprehensive multi-indicator combined system (125, 126).

Future research should prioritize large-scale, prospective, multicenter studies to validate the prognostic value of peripheral blood inflammatory markers and establish standardized assessment protocols. Integrating these markers into comprehensive evaluation systems could improve the precision of disease monitoring, relapse prediction, and therapeutic decision-making. Overall, peripheral blood inflammatory markers offer a practical, accessible, and informative approach with substantial potential to guide prognosis and optimize clinical management in NMOSD, pending further validation.
